# New Insights into the Role and Therapeutic Potential of Heat Shock Protein 70 in Bovine Viral Diarrhea Virus Infection

**DOI:** 10.3390/microorganisms11061473

**Published:** 2023-06-01

**Authors:** Nannan Chen, Tongtong Bai, Shuang Wang, Huan Wang, Yue Wu, Yu Liu, Zhanbo Zhu

**Affiliations:** 1 College of Animal Science and Veterinary Medicine, Heilongjiang Bayi Agricultural University, Daqing 163319, China; nannanchen@byau.edu.cn (N.C.); tongtongbai@byau.edu.cn (T.B.); liuyuyf@163.com (Y.L.); 2 Branch of Animal Husbandry and Veterinary of Heilongjiang Academy of Agricultural Sciences, Qiqihar 161006, China; m13354524787@163.com (S.W.); whuan05172023@163.com (H.W.); 17623409211@163.com (Y.W.); 3 Key Laboratory of Bovine Disease Control in Northeast China, Ministry of Agriculture and Rural Affairs, Daqing 163319, China; 4 Engineering Research Center for Prevention and Control of Cattle Diseases, Daqing 163319, China

**Keywords:** HSP70, Flaviviridae, BVDV, antiviral, potential therapeutic target

## Abstract

Bovine viral diarrhea virus (BVDV), a positive-strand RNA virus of the genus Pestivirus in the Flaviviridae family, is the causative agent of bovine viral diarrhea–mucosal disease (BVD-MD). BVDV’s unique virion structure, genome, and replication mechanism in the Flaviviridae family render it a useful alternative model for evaluating the effectiveness of antiviral drugs used against the hepatitis C virus (HCV). As one of the most abundant and typical heat shock proteins, HSP70 plays an important role in viral infection caused by the family Flaviviridae and is considered a logical target of viral regulation in the context of immune escape. However, the mechanism of HSP70 in BVDV infection and the latest insights have not been reported in sufficient detail. In this review, we focus on the role and mechanisms of HSP70 in BVDV-infected animals/cells to further explore the possibility of targeting this protein for antiviral therapy during viral infection.

## 1. Introduction

Bovine viral diarrhea–mucosal disease (BVD-MD), also known as bovine viral diarrhea (BVD) or mucosal disease (MD), is an infectious disease caused by the bovine viral diarrhea virus (BVDV). This virus can cause acute or chronic infections, leading to persistent infection and weakening the immune system. BVD is considered one of the most costly diseases that can infect a herd, as it can result in significant economic losses [1,2,3,4]. Vaccines effectively prevent BVDV infection; however, achieving adaptive immune protection against BVDV can be challenging due to its diverse nature, ability to infect fetuses, and persistent infections [5]. Heat shock protein 70 (HSP70) is a highly abundant protein that serves as an endogenous immunomodulator of innate and adaptive immune responses. It is also an important anticancer therapy target and prevents immune escape [6,7]. Studies have shown that the HSP70 chaperone molecule plays a unique and important role in interacting with members of the Flaviviridae family, such as the swine fever (CSFV), Zika (ZIKA), and dengue viruses (DENV) [8,9,10]. HSP70 is powerful in regulating oxidative-stress-induced cell damage and death [11]. Given these findings, HSP70 shows promise as a therapeutic target for the development of novel antiviral drugs. However, while there are numerous reports on the antiviral effects of HSP70, research on its anti-BVDV effects still lacks sufficient understanding. Therefore, this paper aims to provide a comprehensive overview of the mechanism and research progress of HSP70 against Flaviviridae, especially BVDV, based on the results of previous research of our team and the latest reports in the field. We hope that this study will provide a reference for future research on the HSP family and flaviviruses.

## 2. Bovine Viral Diarrhea Virus and Its Induced Immunosuppression

### 2.1. BVDV Infection

The Flaviviridae family is responsible for causing severe diseases in both humans and animals. Within this family, there are three different genera: Pestivirus, which includes bovine viral diarrhea virus (BVDV); flaviviruses, which include yellow fever virus (YFV), dengue virus (DENV), and West Nile virus (WNV); and hepatitis viruses, which include hepatitis C virus (HCV) [12]. BVDV is considered one of the most significant economic viruses that affect cattle. However, this pathogen can also infect pigs and various domestic and wild ruminants [13]. Acute BVDV infection is associated with immune dysfunction and can cause peripheral lymphopenia and apoptosis [14]. In addition to causing a range of clinical manifestations, this virus is also a problematic contaminant in laboratory settings [12,15].

### 2.2. BVDV Typing and Its Distribution

**Figure 1:**
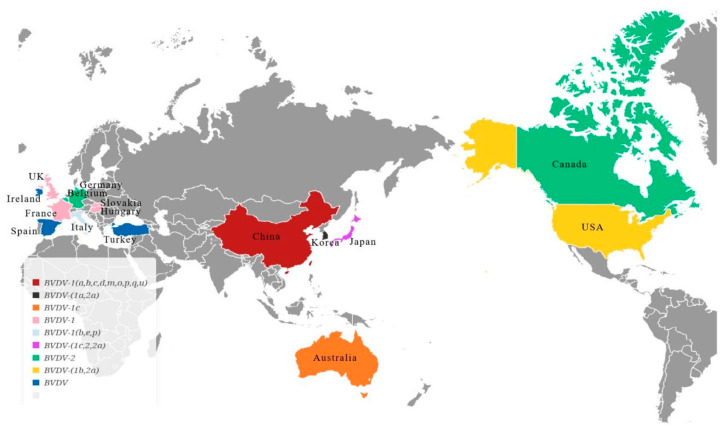
Major countries represented according to the identification of BVDV.

To classify BVDV, scientists observe its effects on cell cultures, categorizing it as cytopathic (CP) or noncytopathic (NCP) [16,17,18,19,20]. BVDV can be classified into three genotypes, namely, genotype 1 (BVDV-1), genotype 2 (BVDV-2), and genotype 3 (BVDV-3), based on the genetic variations in the viral genome [21,22]. BVDV-1 can be divided into 22 gene subtypes (1a~1v) based on 5′-UTR, Npro, and E2 coding sequences. BVDV-2, on the other hand, has four gene subtypes (2a~2d), and BVDV-3 has the subtypes 3a~3d, determined by differences in the secondary structure of the 5′-UTR of the BVDV-2 type. BVDV-1 and BVDV-2 are antigenically distinct; an effective vaccine should always contain both genotypes. The most prevalent subgenotype of BVDV worldwide is BVDV-1b, followed by BVDV-1a and BVDV-1c. In the United States and Japan, BVDV-2a is more common, while in Australia, BVDV-1c is more prevalent. BVDV-1b, 1m, and 1u are the primary subtypes in China. Both BVDV-1 and BVDV-2 can cause severe disease, with BVDV-1 including classical European isolates commonly used for laboratory diagnosis and vaccine production, while the BVDV-2 virus is more pathogenic [21,22,23,24,25,26,27,28], as shown in Figure 1.

### 2.3. BVDV-Induced Immunosuppression in the Body

BVDV initially enters epithelial cells, lymphocytes, and monocytes, replicating and spreading throughout the lymphatic system and compromising the immune systems of infected animals. Studies have shown that B cells, BT cells, monocytes, and macrophages in bovine peripheral blood lymphocytes (PBMCs) can be infected by BVDV [29,30,31]. Many viruses, including BVDV, can hijack the host’s immune response using unique immune evasion strategies to ensure that they successfully replicate and spread from one host to another. BVDV uses several strategies, such as adaptive survival, “hit and run”, and persistent infection, to evade the host’s immune response, which promotes its replication and spread. These strategies also increase the difficulty of detecting new virus strains using the current diagnostic methods. Furthermore, they can lead to the emergence of mutated strains, which may reduce the effectiveness of the current BVDV vaccines. The establishment of persistent infection with BVDV in animal populations relies on the establishment of innate immune tolerance in fetuses carried by pregnant animals, which can lead to the production of PI calves [32].

Our previous study demonstrated that the upregulation of programmed death-1 (PD-1) induces lymphocyte functional failure and suppresses lymphocyte proliferation and apoptosis during acute and chronic viral infections. We examined the mRNA and protein expression of PD-1 and programmed death ligand 1 (PD-L1) in peripheral blood mononuclear cells (PBMCs) infected with BVDV and analyzed the effect of PD-1 blocking on the immune-related function and activity of peripheral blood lymphocytes (PBLs). The results showed that both cytopathic (CP) BVDV (NADL strain) and non-cytopathic (NCP) BVDV (strain KD) infection significantly stimulated the mRNA and protein expression of PD-1 and PDL1. The upregulated expression of PD-1/PD-L1 was accompanied by a decreased proliferation of PBLs and increased apoptosis. Moreover, PD-1 could restore proliferation, inhibit apoptosis, increase IFN-γ production, and reduce the BVDV load. Notably, the PD-1/PD-L1 interaction had a more pronounced effect on the immunoregulation of proliferation suppression induced by CP BVDV infection. Our findings confirmed the crucial role of PD-1 in peripheral blood lymphopenia and apoptosis caused by acute BVDV infection, providing new insights with which to explore the immunosuppressive mechanisms of BVDV or other members of the Flaviviridae and potential therapeutic strategies that could be used to control BVDV infection [14,33].

## 3. Heat Shock Protein 70 and Its Role in Viral Infection

### 3.1. Heat Shock Protein 70 Characteristics

Heat shock proteins (HSP) are also known as stress proteins (SP). The stress response of living organisms can be observed everywhere. It is a series of highly programmed events triggered in biological cells, which play a main role in coping with external environmental changes to protect themselves from damage. As its name implies, the heat shock protein is initially expressed in large quantities after high temperatures and other conditions stimulate the organism. Recent studies have found that many stress conditions can induce the increase in HSP expression in the body, such as viral infection, heavy metals, epoxide inhibitors, various rays, and free radicals [34,35,36]. According to their relative molecular sizes, HSPs can be divided into HSP100, HSP90, HSP70, HSP60, and small HSP families. Among them, HSP70, with a molecular weight of approximately 70 kDa, is the most abundant type of HSP found in various organisms, including plants, animals, and microorganisms. It is highly conserved, and the research on this protein is extensive and in-detailed [37,38]. Heat shock protein 70 (HSP70) is a molecular chaperone that assists in the folding and degrading of proteins and can transport them across biological membranes. It is a stress-induced protein that is overexpressed in various conditions, such as myocardial ischemia/reperfusion (I/R), inflammation, sepsis, diabetes, and cancer [39].

### 3.2. Heat Shock Protein 70’s Role in Viral Replication

Viruses can induce host cells to produce HSP, but they do not express HSP. Many DNA and RNA viruses (e.g., HIV, HSV, VSV, HBV, NDV, SV40, Newcastle disease virus, vaccinia virus, adenovirus, influenza virus, cytomegalovirus, etc.) can induce HSP production. Different types of cells infected with the same virus may result in varying levels of HSP family and HSP expression. This variability may be influenced by factors such as the location of the virus’s early replication, whether in the nucleus or cytoplasm of the host cell [40,41,42,43]. The relationship between virus infection and HSP is complex and diverse. For instance, when the vaccinia virus (VV) infects HeLa cells, it does not produce HSP70, but when HSV-1 and adenovirus (Ads) infect HeLa cells, they express HSP70 at high levels [40,41,42,43]. This diversity is related to various factors such as the structure of the virus, its genetic and infection characteristics, the virus life cycle, and the type of infected host cell.

Viral proliferation depends on the successful recruitment of host cell components for auto-replication, protein synthesis, and virosome assembly. In the generation of viral particles, large amounts of protein are synthesized relatively quickly, potentially causing protein folding to become the limiting step. Therefore, most viruses require cellular chaperones during their life cycles. In addition to their protein-folding problems, viruses must interfere with cellular processes, such as signal transduction, cell cycle regulation, and apoptosis induction, to create a favorable environment for their proliferation and avoid premature cell death. Molecular chaperones are involved in the control of these cellular processes, and some viruses reprogram their host cells by interacting with them. As the most abundant protein in the family, HSP70 plays the role of “guardian” [34,35,36,37,44,45]. The chaperone protein HSP70, which is a key component of the cellular chaperone network, is frequently utilized by viruses. During infection, the production of a significant number of viral proteins can result in cellular stress, leading to an increase in the expression of HSP70. This presents an opportunity for viruses to exploit HSP70 in order to enhance their replication and production [45].

### 3.3. Heat Shock Protein 70 in the Immune Regulation of Antiviral Immunity

HSP70 can perform immunomodulatory effects in viral infection, mediating the immune regulation of interferon and other antiviral substances. Kim et al.’s study revealed that measles virus infection could activate a different interferon release pathway by inducing the differential expression of HSP70 in cell lines or mice. Upon a transcriptional analysis of the infection process, it was discovered that HSP70 mediated the expression of IFN-1, thereby eliminating the need for an increase in its expression. Although the expression of IFN-β is dependent on HSP70, its release is facilitated by toll-like receptors 2 and 4. These processes, which suggest HSP70’s involvement in antiviral activity, differ in their mechanisms for inducing IFN production [46]. Later, in 2013, the same team found that HSP70 could mediate the release of IFN-1. In the early stages of infection, intracellular HSP70 is released into the matrix, leading to increased apoptosis at the time of viral infection. Research conducted on HSP70 knockout mice revealed that HSP70 protein could enhance the clearance of viruses and improve the survival rate of mice. This effect is linked to the expression of IFN-1, which was higher in the normal mice. Consequently, HSP70 exhibits cytoprotective properties while also mediating the inhibition of virus replication [46,47].

### 3.4. Heat Shock Protein 70 as an Adjuvant for Novel Antiviral Vaccines

In antiviral vaccines, HSP70 is used as an adjuvant to improve the release of immunoglobulin and thus improve immunity [48]. In animal experiments, HSP70 obtained from tumors could act as an adjuvant to anti-tumoral vaccines in order to provoke a strong T-cell-dependent antibody effect in humoral immunity [49]. Similarly, the viral peptide complex of HSP70 was also found to stimulate CTL and NK immunity to produce various antiviral effects. Immunizing mice with a fusion protein of HSP70 and HIV-1p24 produced high titers of antibodies against HIV-1p24, while immunizing mice with a non-covalent mixture produced poor results. This outcome may be related to the re-immune response of HSP70 [50]. HSP70 acts as a scaffold in the construction of vaccines. After binding with antigenic peptide, it can become an effective antigen once it enters the immune system. In fact, HSP70 is a highly efficient delivery vector of antigenic peptide.

## 4. Role of Heat Shock Protein 70 in Flaviviridae Viruses Infection

### 4.1. Role of Heat Shock Protein 70 in BVDV Infection

Even though the role of HSP70 in fighting Flaviviridae viruses is significant, its impact on BVDV has not yet been documented, and our study fills this gap [25]. We developed a lentivirus that overexpresses HSP70 and HSP70 siRNA. We examined the impacts of HSP70 overexpression and knockdown on BVDV virus replication at various time points following BVDV (CP/NCP) infection. Our findings demonstrated that, after BVDV infection (CP/NCP), the mRNA and protein levels of both HSP70 and the virus were higher in the overexpression group than in the control group (*p* < 0.05). Additionally, over time, the expression levels of both HSP70 and the virus increased. However, the group with HSP70 siRNA knockdown showed opposite results (*p* < 0.05). Previous research has shown that CP BVDV infection can lead to oxidative stress [11]. Additionally, HSP70 has been identified as having a robust antioxidant function [51]. To better understand the impacts of HSP70 on the regulation of antioxidant genes and oxidative stress during BVDV replication, we treated MDBK cells with HSP70 siRNA and subsequently infected them with BVDV (CP/NCP). We then measured the corresponding oxidative stress markers, antioxidant genes, and enzymes. Our findings revealed that CP BVDV could significantly increase ROS production, while NCP BVDV did not. Furthermore, BVDV stimulates increased MDA levels, decreasing antioxidant genes and enzymes. However, treatment with HSP70 siRNA resulted in a relative reduction in both MDA and ROS production and an alleviation of the decrease in antioxidant enzymes. These results suggest that the role of HSP70 in BVDV infection is closely tied to its antioxidant activity [25].

Extracellular-signal-regulated kinases (ERK) 1/2 are well-known for their essential role in cell signaling. ERK promotes the phosphorylation of cellular substrates, regulating cell growth, differentiation, and stress responses, and can control interleukin-2 (IL-2) production. The MEK/ERK pathway is a vital mechanism for cell survival [24,52,53]. The MEK/ERK signaling pathway is also responsible for the key process of transmitting signals from cell surface receptors to the nucleus. It plays a critical role in controlling various physiological processes, including cell proliferation, migration, and differentiation. When EGF signaling is present, the activated ERK phosphorylates the serine residue at positions 385 and 400 in the linker region of the SBD of HSP70. This results in a phosphorylated HSP70 molecule that assumes an extended conformation. This conformation enhances HSP70’s folding activity and promotes cell proliferation, leading to cancer progression. However, through dephosphorylation, the extended conformation of HSP70 can be converted to a non-extended conformation. A novel inhibitor called VER155008, which targets HSP70, has been found to exert antitumor effects by inhibiting the PI3K/AKT/mTOR and MEK/ERK pathways [54].

Previous studies have indicated that BVDV infection can affect the ERK signaling pathway [55]. It is worth noting that the ERK-dependent phosphorylation of HSP70 facilitated its folding activity and cellular proliferative function [56]. ERK-dependent phosphorylation promotes HSP70 folding activity and cell proliferation [57]. Conversely, the downregulation of HSP70 promotes cell apoptosis [58]. Our previous studies found that PD-1 blockade increased p-ERK levels in mice infected with BVDV [59]. To investigate the impact of HSP-70-mediated ERK regulation on BVDV replication, we infected MDBK cells with BVDV (CP/NCP) after interfering with HSP70 and measured the expression level of ERK. The results showed that the level of p-ERK in the CP-BVDV infection group was significantly lower than that in the control group (*p* < 0.05), but there was no change in the NCP-BVDV group. Therefore, we speculated that BVDV-induced ERK phosphorylation in MDBK cells might be closely linked to HSP70 expression [25].

These findings suggest that HSP70 plays a positive role in regulating BVDV and that inhibiting HSP70 can block BVDV replication and reduce the oxidative stress and ERK phosphorylation caused by BVDV. A broad-spectrum inhibitor of flavivirus infection was identified as a small-molecule inhibitor of HSP70’s ATPase activity [60]. Immunizing pigs with HSP70 fusion protein also promoted lymphocyte proliferation [61]. Our study demonstrated that BVDV replication was inhibited by either the HSP70 inhibitor quercetin or HSP70 knockdown. Thus, HSP70 could be a potential therapeutic target or therapeutic agent against BVDV.

### 4.2. Role of Heat Shock Protein 70 in ZIKV Infection

Zika virus (ZIKV) is a mosquito-borne flavivirus that causes neurological disorders and microcephaly. Pujhari et al. found that inducing HSP70 expression in mammalian cells increased ZIKV production, whereas inhibiting HSP70 activity reduced ZIKV viral RNA production and virion release from the cell. Further studies showed that HSP70 was localized both on the cell surface, where it could interact with ZIKV during the initial stages of the infection process, and intracellularly, where it localized with viral RNA. Blocking cell-surface-localized HSP70 using antibodies decreased ZIKV cell infection rates and the production of infectious virus particles, as did competition with recombinant HSP70 protein. Overall, HSP70 was found to play a functional role in both the pre- and post-ZIKV infection processes affecting viral entry, replication, and egress [10]. Taguwa et al. found that different cytosolic HSP70 isoforms are recruited to ZIKV-induced compartments and are required for virus replication in the pre- and post-entry steps. Drugs targeting HSP70 significantly reduce the replication of different ZIKV strains in human and mosquito cells, including human neural stem cells and a placental trophoblast cell line, at doses without appreciable toxicity to the host cell [62].

### 4.3. Role of Heat Shock Protein 70 in HCV Infection

In a systematic study conducted by Khachatoorian et al. on HSP70 and HCV, it was found that HSP70 inhibited the replication of HCV. It also interacted with the NS5A protein of HCV, which upregulated the expression of HSP70. HSP70 then bound to the viral replication complex, assisted in its assembly, and participated in viral gene replication [63,64]. Another protein in the HSP70 family called HSC70 affects HCV in a manner different from HSP70. The part of HSP70 that binds to NS5A is mainly the amino acid looping region, whereas HCS70 does not bind to it. When the body is infected with HCV, HSC70 is mostly found inside the cells, while HSP70 is mostly found outside the cells. Both HSP70 and HSC70 can inhibit HCV, but HSP70 has greater future potential as a new target for HCV [47,65]. According to Gonzalez et al., HSP70 affects viral proliferation through its interaction with NS5A, a component of the viral replication complex. However, the authors did not analyze its specific effect on viral proliferation [66].

### 4.4. Role of Heat Shock Protein 70 in JEV Infection

Japanese encephalitis virus (JEV) is an enveloped flavivirus and the most common agent of viral encephalitis. In a study on Japanese encephalitis virus (JEV), it was found that the nonstructural protein 5 (NS5) of JEV has type I interferon (IFN) antagonistic properties, which helps the virus to escape the immune response and even promotes viral anti-apoptosis. The upregulation of HSP70 by JEV NS5 not only contributes to type I IFN antagonism but also participates in the anti-apoptotic effects of the JEV NS5 protein by preventing IFN-β-induced p38 MAPK/stat 1-mediated apoptosis [67]. Moreover, the binding of HSP70 to lipid rafts is essential for JEV infection in Huh 7 cells, according to Zhu YZ [68]. JEV typically enters the host cells through receptor-mediated clathrin-dependent endocytosis, which requires HSP70, as noted in a study by Chuang CK [69]. Another study showed that during JEV infection, HSP70 partially colocalizes in the cytoplasm with components of the viral replicase complex, including NS3, NS5, and viral dsRNA. The knockout of HSP70 resulted in a significant reduction in JEV genome replication [70].

Das, S. et al. verified the interaction between HSP70 and JEV using different methods. Indirect immunofluorescence and flow cytometry analysis demonstrated the localization of HSP70 on the Neuro2a cell surface. Co-immunoprecipitation followed by Western blot analysis confirmed the interaction between HSP70 and the JEV-E protein. Furthermore, anti-HSP70 polyclonal antibodies were able to block JEV entry into Neuro2a cells. Additionally, using the bioinformatic tool FTDOCK, docking between the proteins was performed. Amongst the six interacting structural poses studied, one pose involving the RGD motif on JEV-E and leucine (539) on HSP70 displayed stable interaction. These observations indicate that HSP70 is a putative receptor of JEV in Neuro2A cells [71].

### 4.5. Role of Heat Shock Protein 70 in DENV Infection

Dengue virus (DENV) is the causative agent of the most important mosquito-borne viral disease. DENV relies on certain unknown cellular receptors on the host cell surface for viral entry. HSP90 and HSP70 have been identified as participating in DENV entry in human cell lines and monocytes/macrophages, and both heat shock proteins are associated with lipid rafts [70]. Vega-Almeida and colleagues conducted experiments using immunofluorescence and flow cytometry assays. They observed that HSP70/HSC70 and, to a lesser extent, BiP were re-localized to the plasma membrane when cells were under stress conditions, such as DENV infection. By performing binding and infection assays independently, they found that all four proteins participated in both processes but to differing extents: HSP70/HSc70 was the most critical component, while ERp44 was less important [72]. In addition, HSP70 can facilitate DENV virus propagation by inhibiting the type 1 interferon response [73]. Howe et al. further demonstrated the importance of HSP70i in the pathogenesis of the dengue virus. A small-molecule inhibitor of heat shock protein 70 was shown to exhibit anti-dengue virus activity, confirming HSP70’s status as a host antiviral target [74].

### 4.6. Role of Heat Shock Protein 70 in CSFV Infection

Classical swine fever is a disease listed by the World Organization for Animal Health, and it is caused by the classical swine fever virus (CSFV). Chengcheng et al. studied the interaction between CSFV and HSP70. They found that CSFV infection results in the elevated expression of HSP70 in host cells, which is attributed to the interaction between HSP70 and NS5A [8]. In the same year, Xu Q et al. also conducted relevant research. Prokaryotic plasmids containing the envelope glycoprotein E0 gene of the classical swine fever virus (CSFV) and/or the HSP70 gene of Haemophilus parasuis were created and expressed in Escherichia coli Rosetta 2 (R2). After purifying the fusion proteins, groups of Balb/c mice were immunized with each of them. Serum samples were collected from the mice seven days after the third immunization. The immune effects were determined via an enzyme-linked immunosorbent assay and flow cytometric analyses. The combination of the E0-HSP70 fusion protein and E0+HSP70 mixture resulted in a significant improvement in the production of E-specific antibodies and increased levels of CD4+ and CD8+ T cells, as well as the release of interferon-γ. These findings suggest that HSP70 can significantly enhance the immune effects of the envelope glycoprotein E0 of CSFV [75]. Reviewing the literature, we found that CSFV and HSP70 studies are still rarely reported, and the research mechanism is inadequate.

### 4.7. Role of Heat Shock Protein 70 in WNV Infection

West Nile virus (WNV) is a member of the Flavivirus family and induces febrile illness, sporadic encephalitis, and paralysis. During West Nile virus (WNV) infection, multiple isoforms of HSP70 are upregulated in cells, and studies have shown that HSP70 interacts with WNV Cp [76]. Oh et al., using WNV Cp as bait for a yeast two-hybrid assay, found that HSP70 interacted with WNV Cp. After a deletion analysis of HSP70, it was discovered that the substrate-binding domain of HSP70 can bind to WNV Cp. In addition, the cytotoxic effect of WNV Cp in 293T cells was prevented by ectopic HSP70, suggesting a negative regulatory role of HSP70 in WNV Cp [77].

### 4.8. Role of Heat Shock Protein 70 in EBOV Infection

Ebola virus (EBOV) infection results in severe disease and, in some cases, lethal hemorrhagic fever. EBOV has a negative-sense RNA genome encapsidated by the virally encoded nucleoprotein (NP). Garcia-Dorival et al. investigated the interaction between cellular chaperones (including HSP70) and EBOV proteins and the identification of therapeutic targets. High-affinity co-immunoprecipitation coupled with a label-free mass-spectrometry-based approach was used. By utilizing conservative selection criteria, the scientists identified around 150 cellular proteins having a high probability of interacting with NP. These included the heat shock protein 70 (HSP70) and members of the protein chaperone family. The inhibition of HSP70 function resulted in the degradation of NP, suggesting a role of HSP70 in modulating the stability of the protein [78].

### 4.9. Role of Heat Shock Protein 70 in TMUV Infection

HSP70 also plays an important role in poultry viruses. Tianbusovirus (TMUV) is an avian-derived flavivirus prevalent in ducks and geese, causing huge economic losses for the waterfowl industry. It was discovered that HSP70 plays an important role in the later stages of the TMUV life cycle, including viral replication, assembly, and release. The inhibition of HSP70 expression can significantly reduce TMUV-induced apoptosis [79]. In conclusion, HSP70 plays a significant role in the infection of Flaviviridae viruses and can be utilized as a target protein for the development of anti-Flaviviridae virus treatments. However, it can be seen from the above data that there are few in vivo studies on each virus, and the effect of HSP70 on the above viruses requires further research, as indicated in Table 1.

**Table 1:** Mechanism of action of HSP70 with Flaviviridae viruses.

Virus	In Vitro/In Vivo Study	Mechanism	Reference
BVDV	In vitro	HSP70 promoted BVDV replication in MDBK cells, and the inhibition of HSP70 impeded BVDV replication. The regulation of HSP70 expression has the potential to impact oxidative stress and ERK phosphorylation induced by BVDV.	[25]
ZIKV	In vitro	HSP70 mediated ZiKV’s entry, replication, and expulsion from host cells.	[10]
	In vitro and in vivo	Different cytosolic HSP70 isoforms are recruited to ZIKV-induced compartments and are required for the virus to replicate during pre- and post-entry steps.	[62]
WNV	In vitro	The upregulation of numerous subtypes of HSP70 led to increased viral replication, and HSP70 was found to interact with WNV Cp.	[76]
	In vitro	HSP70 functions as a negative regulator of the West Nile virus capsid protein through direct interaction.	[77]
HCV	In vitro	The infection of host cells with HCV can increase the expression of HSP70, which can bind to the viral replication complex and facilitate the assembly and replication of viral genes.	[63,64]
	In vitro	The binding site for NS5A/HSP70 was determined to be a hairpin moiety at the C-terminus of NS5A domain I and showed a corresponding cyclized polyarginine-tagged synthetic peptide (HCV4) that significantly blocks virus production.	[65]
	In vitro	HSP70 interacts with NS5A, one of the components of the viral replication complex, thereby affecting the proliferation of the virus.	[66]
CSFV	In vitro	The data suggest that HSP70 is critical in the viral life cycle, particularly during the virus RNA replication period.	[8]
	In Vivo Study	HSP70 fused with the envelope glycoprotein E0 of the classical swine fever virus, enhancing immune responses in Balb/c mice.	[75]
JEV	In vitro	The upregulation of HSP70 by JEV NS5 plays a dual role by contributing to the antagonism of type I IFN and participating in the anti-apoptotic effect of JEV NS5. This is achieved by preventing p38 MAPK/Stat1-mediated apoptosis induced by IFN-β.	[67]
	In vitro	The binding of HSP70 to lipid rafts is necessary for JEV infection in Huh7 cells.	[68]
	In vitro	JEV usually enters host cells through receptor-mediated clathrin-dependent endocytosis, which requires HSP70/Hsc70 to be composed of at least three subtypes (B, C, and D).	[69]
	In vitro	During JEV infection, HSP70 in the cytoplasm colocalized with components of the viral replicase complex (NS3, NS5, and viral dsRNA). Knockout of HSP70 resulted in a significant reduction in JEV genome replication.	[70]
	In vitro	Heat shock protein 70, in Neuro2a cells, is a putative receptor of the Japanese encephalitis virus.	[71]
	In Vivo Study	Immunizing pigs with HSP70 fusion protein can promote lymphocyte proliferation.	[59]
DENV	In vitro	HSP90 and HSP70 are involved in the entry of the dengue virus as receptor complexes in human cell lines and monocytes/macrophages.	[70]
	In vitro	HSP70 helps the virus to reproduce by inhibiting the type 1 interferon response.	[70]
	In vitro	HSP70/HSc70 is the most critical component of DENV infection.	[72]
	In vitro	The use of JHSP70 inhibitors was shown to block dengue virus activity, indicating that HSP70 can be exploited as a host antiviral target.	[73]
EBOV	In vitro	The binding of HSP70 to the EBOV protein promotes its stability.	[78]
TMUV	In vitro	HSP70 plays an important role in the TMUV life cycle (virus replication, assembly, and release). The inhibition of HSP70 expression significantly reduces TMUV-induced apoptosis.	[79]
Flavivirus	In vitro and in vivo	Small-molecule inhibitors of HSP70 ATPase activity can be used as broad-spectrum inhibitors against flavivirus infection.	[59]

### 4.10. Antiviral Effects of the Heat Shock Protein 70 Inhibitor Quercetin

#### 4.10.1. The HSP70 Inhibitor Quercetin in Flaviviridae Virus Infection

RNA viruses are known to mutate quickly, creating many new drug-resistant strains that render many drugs and vaccines used against the virus ineffective. Therefore, searching for antiviral target molecules in host cells could help to control the growth and reproduction of various persistent viruses during infection, providing a new approach to antiviral treatment. HSP70 is recognized as an important target for anticancer therapy and the prevention of immune escape and plays a major role in viral replication in the Flaviviridae family. Quercetin is widely distributed in the plant kingdom and is one of the well-known types of plant metabolites. It is a flavonol compound with various biological activities [16]. Quercetin has a stable chemical structure and water-soluble derivatives with strong anti-inflammatory and immunomodulatory effects. It is also a natural, safe antioxidant with minimal side effects [23,24,80]. It is often used in healthcare products to increase immunity or prevent infection and can be widely used in medicine and animal feeds. It has broad clinical application prospects [81]. Many studies have shown that quercetin can inhibit the expression of HSP70 [8,82,83,84]. Importantly, quercetin has a significant inhibitory effect on Flaviviridae viruses [23,85,86,87,88,89,90,91,92,93,94,95], as shown in Table 2.

**Table 2:** Quercetin’s applications against Flaviviridae viruses.

Virus	Mechanism	Reference
DENV	Quercetin has the best binding energy for NS2B-NS3 protease.	[85]
	Quercetin interacts with the biological molecules of dengue virus serotype 3 NS2B-NS3 protease to inhibit dengue virus.	[86]
	Quercetin showed the most significant preventive effect against DENV infection. In addition, quercetin also showed the strongest binding affinity for the DENV membrane receptor TIM-1 protein in molecular docking analysis.	[87]
	Quercetin affects DENV-2 virus replication but does not affect DENV-2 attachment to host cells.	[88]
	Quercetin inhibits viral replication in vivo and in vitro in a dose-dependent manner.	[90]
Coinfection wtith COVID-19 and dengue fever	Quercetin inhibits cytokine release through NF-κB, IL-17, and toll-like receptor signaling pathways, alleviates over-immune responses, and eliminates inflammation.	[89]
ZIKV	Quercetin prevents the internalization of the virus particles to the host cell, preventing the entry of ZIKV.	[91]
	Quercetin targets viral replication and almost completely inhibits Zika virus RNA production.	[93]
JEV	Quercetin reduced the JEV RNA copy number.	[92]
HCV	Quercetin inhibits the activity of the viral protease non-structural protein 3 (NS3), effectively targeting and reducing HCV RNA and resulting in reduced HCV replication.	[23]
	Quercetin decreased the production of reactive oxygen and nitrogen species (ROS/RNS) and lipid peroxidation induced by HCV and inhibited HCV replication. Quercetin decreased the increased expression of diglyceride acyltransferase 1 (DGAT1) mRNA after viral infection and inhibited HCV genome replication. Quercetin can affect the morphogenesis of infectious particles and reduce HCV-specific infectivity. The integrity of virions is affected when applied directly to HCV particles, hindering the localization of the HCV core protein in LDs.	[94]
EBOV	Quercetin blocks Ebola virus infection by inhibiting the inhibitory function of VP24 interferon.	[95]

#### 4.10.2. Quercetin Suppresses HSP70’s Effects in Viral Infection

Research studies have reported that quercetin can inhibit the translation of the swine fever virus (CFSV) and hepatitis C virus (HCV). This is achieved by blocking the translation process mediated by Non-Structural Protein 5A (NS5A) and the Internal Ribosome Entry Site (IRES), as well as inducing the expression of Heat Shock Protein 70 (HSP70) [8,82,96,97]. However, the NS5A protein is also an important functional protein in BVDV virus replication. Our recent study demonstrated that quercetin could inhibit HSP70 and thus BVDV replication in a dose-dependent manner, being closely related to the antioxidant function of HSP70 and quercetin. However, the question of whether HSP70 interacts with BVDV-NS5A still needs to be determined. Through in vivo testing using a BVDV model in mice, it was observed that quercetin significantly reduced the BVDV virus titer, lessened oxidative stress, promoted the expression of antioxidant genes, and alleviated immune suppression and tissue damage. However, further validation is required to determine whether quercetin achieves these effects by regulating HSP70 to inhibit BVDV [25].

Gao et al. found that HSP70 was significantly increased in lungs infected with the porcine reproductive and respiratory syndrome virus (PRRSV), indicating that HSP70 may play a potential role in PRRSV infection. Subsequently, the authors studied the specific role of HSP70 in PRRSV infection and observed that the expression of HSP70 was induced upon PRRSV infection. Using quercetin, an HSP synthesis inhibitor, or small interfering RNAs (siRNA), the downregulation of HSP70 reduced the viral protein level and production. Notably, heat shock treatment could attenuate these inhibitory effects on PRRSV infection. In addition, HSP70 was found to colocalize with the viral double-stranded RNA (dsRNA), and its knockdown decreased the dsRNA levels. These findings indicate that HSP70 forms the viral replication and transcription complex (RTC), ultimately impacting viral replication [98]. In addition, regarding anti-JEV, Weng reported that HSP70 downregulation induced by quercetin significantly recovered the IFN-β-induced apoptosis of NS5-expressing cells, correlated with the increase in the phosphorylation of ERK2, p38 MAPK, and STAT1 [67].

In plants, the HSP70 protein functions similarly to its animal counterpart. Quercetin was shown to downregulate the expression of HSP70 protein in plants by inhibiting NbHSP70er-1 and NbHSP70c-A, which can lead to the inhibition of tobacco mosaic virus (TMV) and the proliferation of potato virus [99]. In addition, cell membrane HSP70 is involved in multiple aspects of tomato dwarf virus (TBSV) replication, such as regulating the subcellular localization and membrane insertion of viral replication proteins, as well as facilitating the assembly of viral replicase. Treatment with quercetin led to the downregulation of HSP70 protein expression in cells, resulting in a significant reduction in the RNA proliferation of TBSV [100]. In conclusion, targeting the molecular chaperone HSP70 and inhibiting HSP70 with its specific inhibitors, thus affecting viral proteins or viral RNA replication, can be an effective anti-BVDV strategy, as shown in Table 3.

**Table 3:** Quercetin inhibits HSP70 antiviral.

	Mechanism	Reference
BVDV	Quercetin inhibits HSP70 and thus BVDV virus replication (in the early stage of BVDV infection)	[25]
CFSV/HCV	Quercetin inhibits viral translation by blocking the induction of the host protein (NS5A) and HSP70	[8,82]
PRRSV	Quercetin inhibits HSP70 and reduces porcine reproductive and respiratory syndrome virus replication in vitro.	[98]
JEV	Quercetin-induced downregulation of HSP70 led to a significant recovery of IFN-β-induced apoptosis in NS5-expressing cells. An increase in the phosphorylation of ERK2, p38 MAPK, and STAT1 accompanied this process.	[67]
Plant virus	Quercetin inhibits tobacco mosaic virus (TMV) by inhibiting NbHSP70er-1 and NbHSP70c-A and downregulating HSP70 protein expression.	[99]
	Quercetin can inhibit the proliferation of the potato disease virus.	[99]
	Quercetin treatment could downregulate the expression of HSP70 protein in cells and significantly reduce the RNA proliferation of tomato dwarf virus (TBSV).	[100]

## 5. Conclusions and Future Perspective

BVD infection poses a continuing threat to the health and life of cattle. Vaccination is considered a biosafety measure. For decades, several classical weak and inactivated BVDV vaccines have been demonstrated to trigger and induce certain levels of humoral and cellular immune responses. However, the emergence of new strains affects the efficacy of these conventional vaccines. Similarly, no antiviral products are currently licensed for cattle, indicating an urgent need for a commercial product that can be used to effectively prevent or treat BVDV [101]. Heat shock proteins are stress proteins with highly conserved amino acid sequences and have various protective functions, such as molecular chaperone, anti-apoptosis, and immune regulation functions. As the most conserved protein in the heat stress protein family, HSP70 protects the body from adverse environments and plays important roles in bacterial and viral infections [34,35,36,102]. The HSP70 family members play various roles in viral infections. These include (1) supporting the virus during entry by forming complexes on host cell surfaces; (2) supporting viral replication through direct interaction with viral polymerase; (3) assisting in the assembly of the viral capsid, with viruses also relying on HSP70 to fold their proteins and increase their survival chances in unfavorable host conditions; and (4) inhibiting the interferon response so as to help the virus to reproduce. Inhibitors targeting HSP70 have shown promise as effective therapeutic options against viral infections [76].

Since the virus’ replication cycle is closely related to the metabolic process of the host normal cells, inhibiting the virus’ reproduction will inevitably lead to the damage of uninfected cells, which is also the reason for the slow development of antiviral drugs. At the same time, the existing antiviral drugs have some defects, such as their high toxicity and side effects, tendency towards drug resistance, and inability to completely remove the virus. Therefore, developing new targeted antiviral drugs without side effects is the key to research and development. In recent years, medicinal plants and their bioactive metabolites have emerged as effective and alternative antiviral active agents [16,81]. Numerous studies have shown that some plant metabolites can impede viral replication without affecting host physiology or have limited side effects [23,80]. Due to its powerful therapeutic properties, multi-targeted efficacy, and low toxicity, the flavonoid quercetin is vital for treating diseases [103,104,105]. Importantly, quercetin, as an inhibitor of HSP70, inhibited BVDV replication while inhibiting HSP70 [25]. Therefore, quercetin, as an inhibitor of HSP70, can be used as a target drug against BVDV. Our study was conducted only on live mice, and quercetin has not been tested in clinical settings. While quercetin is commonly used, its effectiveness in treating BVDV in cattle has not been studied in clinical trials. We will continue to study the relationship between quercetin and HSP70, as well as that between quercetin and BVDV, in order to explore new directions and make progress in identifying drugs that can effectively target BVDV.

Although there are many reports on the antiviral properties of HSP70, there are still few reports on the anti-BVDV properties of HSP70. We reported on the mechanism of replication between HSP70 and BVDV for the first time. However, this research is still in its infancy, and many complex mechanisms have not been thoroughly studied; thus, more in-depth research and discussion are needed. Not only does HSP70 have different effects, but the myriad of effects exhibited may also act synergistically. From a future perspective, the development of HSP70 inhibitors and promoters could provide a new way to combat viral or bacterial infections. We recommend conducting further research to reveal more effective HSPs and their combinations that play a role in antiviral and inflammatory disorders, autoimmune diseases, cancer, and other diseases. In addition, the development of HSP70 inhibitors could star with traditional Chinese medicine or plant active substances. The use of bioactive compounds to regulate basic mechanisms, such as immunity or cell survival, renders these compounds particularly meaningful as targeted drugs or adjuvants for preventing and treating various diseases.

## Data Availability

Not applicable.
